# Input Shape Effect on Classification Performance of Raw EEG Motor Imagery Signals with Convolutional Neural Networks for Use in Brain—Computer Interfaces

**DOI:** 10.3390/brainsci13020240

**Published:** 2023-01-31

**Authors:** Emre Arı, Ertuğrul Taçgın

**Affiliations:** 1 Department of Mechanical Engineering, Faculty of Engineering, Marmara University, Istanbul 34840, Turkey; 2 Department of Mechanical Engineering, Faculty of Engineering, Dicle University, Diyarbakır 21280, Turkey; 3 Department of Mechanical Engineering, Faculty of Engineering, Doğuş University, Istanbul 34775, Turkey

**Keywords:** brain–computer interface (BCI), deep learning, EEG motor imagery, classification, input shape, raw data

## Abstract

EEG signals are interpreted, analyzed and classified by many researchers for use in brain–computer interfaces. Although there are many different EEG signal acquisition methods, one of the most interesting is motor imagery signals. Many different signal processing methods, machine learning and deep learning models have been developed for the classification of motor imagery signals. Among these, Convolutional Neural Network models generally achieve better results than other models. Because the size and shape of the data is important for training Convolutional Neural Network models and discovering the right relationships, researchers have designed and experimented with many different input shape structures. However, no study has been found in the literature evaluating the effect of different input shapes on model performance and accuracy. In this study, the effects of different input shapes on model performance and accuracy in the classification of EEG motor imagery signals were investigated, which had not been specifically studied before. In addition, signal preprocessing methods, which take a long time before classification, were not used; rather, two CNN models were developed for training and classification using raw data. Two different datasets, BCI Competition IV 2A and 2B, were used in classification processes. For different input shapes, 53.03–89.29% classification accuracy and 2–23 s epoch time were obtained for 2A dataset, 64.84–84.94% classification accuracy and 4–10 s epoch time were obtained for 2B dataset. This study showed that the input shape has a significant effect on the classification performance, and when the correct input shape is selected and the correct CNN architecture is developed, feature extraction and classification can be done well by the CNN architecture without any signal preprocessing.

## 1. Introduction

Brain–computer interfaces enable the use of an external device by collecting and processing brain signals in various online or offline methods. To collect brain signals, electrocorticograms (ECoG), magnetoencephalography (MEG), electroencephalography (EEG), positron emission topography (PET), local field potentials and action potentials, functional magnetic resonance imaging (fMRI), and near-infrared spectral imaging (NIRS) methods are used. The distinctive features of these collected signals are obtained by different signal processing techniques. These distinguishing features are classified and integrated into a control system or used as a control signal by connecting to a device provided to fulfill the desired purpose.

Brain–computer interfaces are intensively researched in the fields of disease detection, entertainment, education, marketing, games, medical devices and equipment, robotics and physiotherapy, and real-time applications have tried to be developed [1,2,3,4,5,6,7,8,9,10,11,12,13]. One of the most preferred signal types for brain–computer interfaces is EEG signals. It is often preferred because it is mobile, cheaper and can be applied faster than other signal acquisition methods. In addition, many different BCI paradigms such as visual evoked potentials (VEP), steady-state visual evoked potentials (SSVEP), motion-onset visual evoked potentials (moVEP), P300 evoked potentials and motor imagery (MI) have been developed and applied with EEG signals [14,15]. In the datasets used in this study, individuals generated motor imagery signals by imagining that they performed a movement (such as hand, arm movements, etc.) without physically moving, and these signals were used for classification. For the classification of EEG signals, machine learning and deep learning models are used together with traditional signal processing methods.

Classification processes are carried out by using signal processing and machine learning models, where distinctive features are extracted manually from EEG signals, or by using deep learning models, where feature extraction is done automatically by the model. Researchers have used signal processing methods such as wavelets [16,17], short-time Fourier transforms [18,19], wavelet packet decomposition [20], continuous wavelet decomposition [21], common spatial pattern [22,23,24], filter bank common spatial pattern [25], fast Fourier transforms [26], and Choi–Williams distribution transform [15]. As machine learning and deep learning methods, support vector machine [25], random forest [27], linear discriminant analysis [28], autoencoders [19], artificial neural network, deep belief network, recurrent neural network and convolutional neural network models [29] have been developed and used. In addition, hybrid models consisting of combinations of these methods and models have also been designed. The purpose of these methods and models is to analyze the EEG signals correctly and to extract the necessary distinguishing features. By using these manually or automatically extracted features, it is aimed to classify the brain signals correctly.

While performing feature extraction processes, time, frequency and space domains are studied and temporal and spatial features of EEG signals are extracted. As the classifier models are trained with EEG signals, they are also trained with images obtained after the signals are converted into 2D images [15].

With all the above-mentioned methods, the main goal is to identify the distinctive features of the EEG signals and to classify them with a high classification accuracy. In addition, the designed methods and models are expected to be fast, highly accurate, robust and reliable systems. These are very important requirements for real-time brain–computer interface systems to be developed.

Signal preprocessing applied to EEG signals, which are very sensitive and difficult to analyze and understand, are based on a high level of user knowledge and experience [30,31,32,33,34]. In addition, by trying many different processes, much time and energy is spent choosing the right method [31,35]. Moreover, while these processes applied to the EEG signals can reveal the distinctive features in the signals, they can also cause the loss of these features [31,36,37].

In [32], the researchers explained that many feature extraction methods depend on user knowledge and experience and, as such, these methods may limit the success of the model. They also stated that detecting the right features requires extensive experience and observation, which requires a large amount of time and effort. They explained that high classification success can be achieved, and time and effort can be saved, with feature extraction processes performed automatically by neural networks. In [31], the researchers developed a deep learning network model that performs artifact removing for EEG signals and compared it with different methods. As a result of this comparison, they showed that some traditional models cause data loss and revealed that the model they developed is faster than other models.

Considering all these factors, no preprocessing was applied in this study and training and classification were made with raw data. The way the data used during the training is one of the important factors affecting the success of the model [29]. In our literature review, we observed that researchers use different input shapes in their studies [7,14,19,21,29,30,32,33,36]. We could not find a specific study on the effect of input shapes on classification performance. This shortcoming was one of the inspirations for this study. In this study, the effects of different input shapes on system performance were investigated during the training of EEG motor imagery data with CNN models, which has not been specifically examined before.

In classifications made using machine learning and deep learning, EEG signals are usually preprocessed, the distinctive features of the signals are extracted, the developed models are trained and classifications are made. Studies have shown that Convolutional Neural Networks give better results than other models [29].

**Figure 1:**
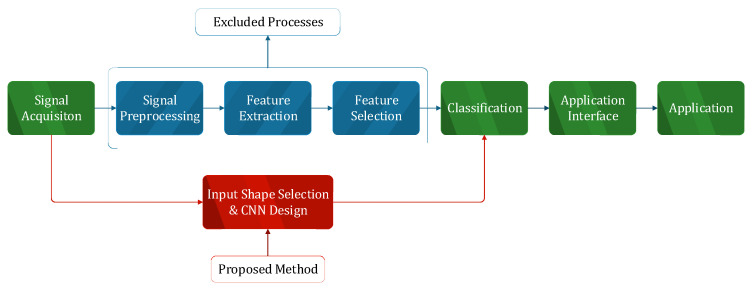
Proposed BCI Processes.

While developing brain–computer interfaces, traditionally, there are 7 stages: signal acquisition, signal preprocessing, feature extraction, feature selection, classification, application interface and application. In this study, a leaner and faster BCI development process is proposed by excluding the signal preprocessing phase using raw EEG signals and feature extraction and feature selection phases with the CNN model design. This proposed methodology is shown in Figure 1.

Researchers have started to train and classify their models using raw data and have achieved remarkable results. In [30], researchers developed a CNN model using raw EEG signals. They stated that using all EEG channels in the dataset to train their model is computationally demanding and includes irrelevant features. In order to avoid this situation and to create a simpler classifier, combinations containing different numbers of EEG channels were tried. They also tried different data augmentation methods to increase the success of the classifier. In their results, they explained their optimal channel configurations and data augmentation methods. In [38], the authors developed a CNN model using separated temporal and spatial filters and raw EEG signals. In that study, they searched for minimal electrode pairs with which they could achieve high classification success and aimed to accelerate clinical applications by simplifying BCI designs. They stated that preprocessing methods used for EEG signals can increase the signal-to-noise ratios and classification success of nonstationary EEG signals, but this is not necessary [38]. In addition, the researchers used raw EEG data in their studies and achieved good results [39,40,41,42]. The promising results obtained from previous studies with raw EEG data [30,32,33,38,39,40,41,42] are one of the reasons that encouraged us to create a model using raw data.

Since EEG signals are nonstationary and show variable characteristics depending on time, covariate shifts occur, and it is very difficult to classify these signals with high performance. The fact that input data distributions vary from person to person, and even in different sessions for the same person, makes it very difficult to develop real-time adaptive systems [43]. Various methods have been developed to prevent the covariate shift effect in studies using machine learning, signal preprocessing and feature extraction methods. In [43], authors developed an exponentially weighted moving average model to detect covariate shifts and designed an ensemble classifier. They updated their model by adding new classifiers to the ensemble that take into account the changes in the input data distribution and estimated shifts over time, and then they compared their method with different studies in the literature.

Feature distributions of training and test sets can be analyzed with density ratio estimation approaches [43]. Some of these approaches are least-squares importance fitting, the Kulback–Leibler importance estimation procedure [44] and kernel mean matching [45].

In [46], the authors aimed to identify the most robust spatial filtering approach using a calibration dataset and a test dataset. They also examined performance variations by applying Stationary Subspace Analysis (SSA). They showed that, among the Common Spatial Pattern (CSP), Filter Bank Common Spatial Pattern (FBCSP), Filter Bank Common Spatial Pattern Time (FBCSPT), Source Power Co-modulation (SPoC), Spectrally Weighted Common Spatial Patterns (SpecCSP), and Surface Laplacian (SLap) methods, the FBCSP and PBCSPT methods are the most robust approaches against feature covariance shift. In addition, after applying the SSA method, they achieved higher accuracy values in both datasets.

In deep learning methods, including our CNN model, learning takes place layer by layer, and the output of each layer becomes the input of another layer. Changes that occur in the input distributions during this type of learning cause a covariate shift in the model, and the hidden layers try to adapt to the new distribution. This slows down the training and makes it very difficult to train models with saturating nonlinearity [47]. In [47], the researchers developed the batch normalization layer to overcome this problem. Using this layer, each batch is normalized to have zero mean and unit variance. In this way, each batch is centered around the zero value and it is ensured that each feature value remains on the same scale.

In this study, among the deep learning classification methods mentioned above, two different CNN models consisting of the same layers and parameters were developed for 2D and 3D data structures. BCI Competition 2A and BCI Competition 2B datasets were used to train and evaluate these CNN models. The data used were transformed into eight different input shape structures and the models were fed with these structures. Training and classification processes were performed separately for each input shape, and accuracy values for each subject, average training accuracy values and epoch times were obtained. These obtained values are given in tables in the Results section. Confusion matrix values and training and validation graphs are shown for each input shape and, finally, comparisons of the obtained results are made. In the Discussion section, the pros and cons of these designed input shape structures that can be used in brain–computer interface designs are discussed. The aim of this study is to investigate the effect of input shapes on classification and to propose classification methods using raw data that will save researchers time and effort in classifying noisy EEG signals, which are very difficult to train and classify [31,32].

## 2. Dataset and Methods

Graz BCI-IV-2A and BCI-IV-2B datasets [48], which are two of the most widely used datasets for classification of EEG motor imagery signals, were used in this study. There are four different classes in the BCI-IV-2A dataset: left hand, right hand, both feet and tongue. These data were collected from 9 different healthy individuals with 22 EEG channels at a sampling rate of 250 Hz and included 288 trials and 5184 attempts. The dataset contains a different set for training and a different set for testing.

In the BCI-IV-2B dataset, there are two classes: left hand and right hand. These data were collected from 9 different healthy individuals with 3 EEG channels at a sampling rate of 250 Hz and included 280 trials and 6520 attempts. The dataset contains a different set for training and a different set for testing. In order to make an accurate comparison of the BCI-IV-2B dataset with the BCI-IV-2A dataset, only the left hand and right hand classes from the 2A dataset were used.

### 2.1. Input Shape

**Figure 2:**
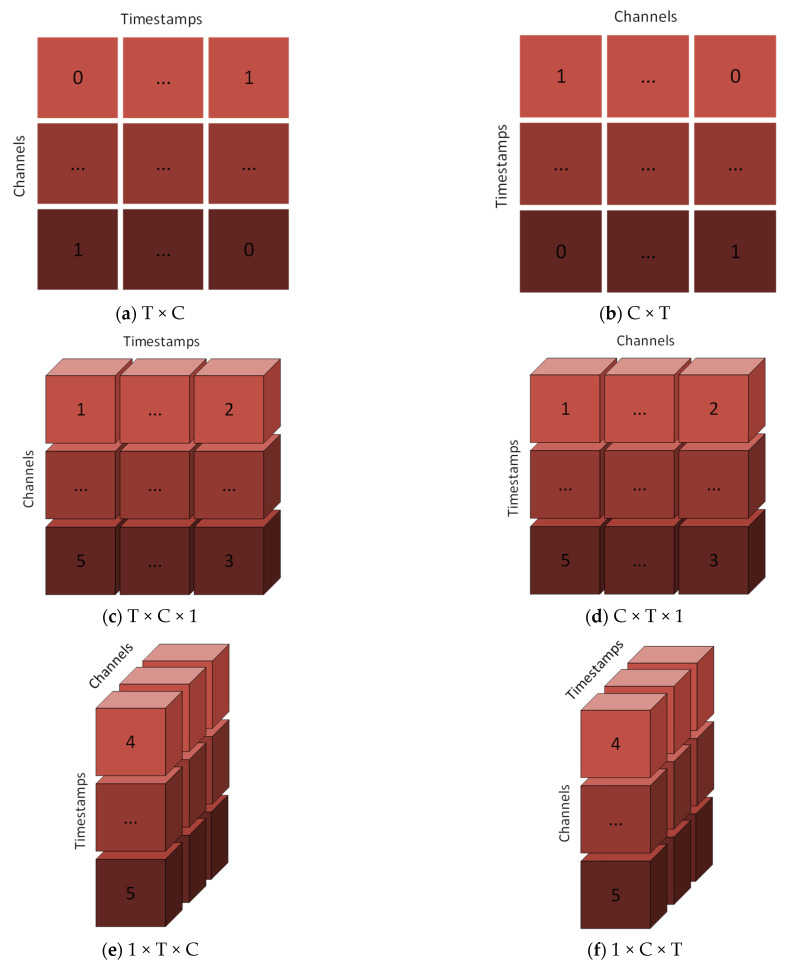

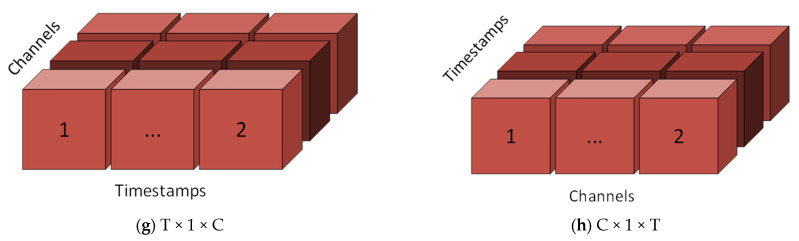
Input Shape Designs.

Input shape structures are one of the most important parameters needed for CNN model designs to achieve high classification accuracies, and researchers have used many different input shape structures in their previous studies [29]. In this study, it was investigated whether faster and higher classification success could be achieved by designing different input shape structures, and these input shape structures were compared with each other. The signals in these datasets have been converted into 2D and 3D structures with 8 different input shapes for each dataset. The transformed data for each dataset is shown in Figure 2.

The purpose of these transformations is not only to train the model with 2D data dimensions, but also to train the model with both 2D and 3D data dimensions to find temporal and/or spatial features in EEG signals.

To fulfill this purpose, each trial is structured according to the height, width and channel (H × W × C) parameters of the library used. Height represents the horizontal axis, width represents the vertical axis and channel represents the third axis. These structures are matrices formed as T × C, C × T, T × C × 1, C × T × 1, 1 × T × C, 1 × C × T, T × 1 × C, C × 1 × T, where C represents the number of EEG channels, and T represents the number of timestamps.

The aim of these different 2D and 3D matrix structures is to examine the effects of shape changes, total number of parameter changes, complexity of calculations on classification speed, robustness and accuracy. The total number of parameter changes is given in Equations (1)–(3).

(1) $$W_{c}=K^{2}\times C\times N\;$$

(2) $$B_{c}=N$$

(3) $$P_{c}=W_{c}+B_{c}$$

where $W_{c}$ is number of weights of the convolutional layer, K is the size of kernels used in the convolutional layer, C is the number of channels of the input, N is the number of kernels, $B_{c}$ is the number of biases of the convolutional layer and $P_{c}$ is the number of parameters of the convolutional layer.

(a) T × C

With this 2D structure, timestamps data are placed on the horizontal axis and EEG channel data is placed on the vertical axis.

(b) C × T

With this 2D structure, EEG channel data are placed on the horizontal axis and timestamps are placed on the vertical axis.

(c) T × C × 1

This 3D structure was created by adding the third axis to the T × C matrix structure. With this structure, CNN models that use 3D matrices as inputs can be used.

(d) C × T × 1

This 3D structure was created by adding the third axis to the C × T matrix structure. With this structure, CNN models that use 3D matrices as inputs can be used.

(e) 1 × T × C

With this 3D structure, the horizontal axis is arranged to contain a single column and data from a single EEG channel data are placed in this column. Afterwards, the remaining EEG channels as the third dimension were placed on this axis.

(f) 1 × C × T

With this structure, a single column is placed on the horizontal axis and timestamps data are placed in this column. The remaining timestamps were placed in the third dimension and it was aimed to find the relationships in this way.

(g) T × 1 × C

With this 3D structure, the vertical axis has been arranged to contain a single row, and the data of all EEG channels have been added to this row. Afterwards, the data of the remaining EEG channels were added as the third dimension.

(h) C × 1 × T

With this structure, timestamps data were arranged in a single row on the vertical axis and the remaining timestamps data were added as the third dimension.

### 2.2. Proposed CNN Model

**Figure 3:**
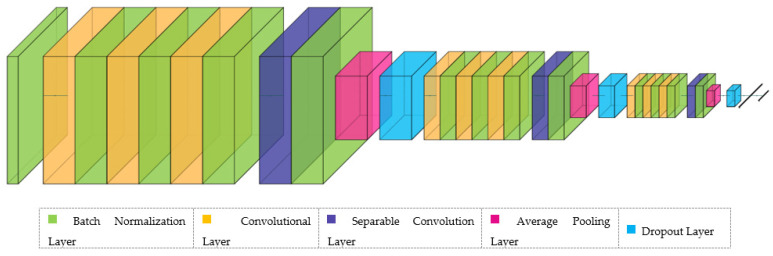
Developed CNN Model Architecture.

Two Convolutional Neural Network (CNN) models were created to train and classify the selected datasets. These models have same layers and parameters and have the architecture needed to train 2D and 3D data. Signal preprocessing methods used in the vast majority of studies in this field were not used in this study, and training and classification were made with raw data. It is predicted that the filters used for different wavelengths can make the distinguishing features blurrier in very sensitive, complex and difficult-to-understand EEG signals, and feature extraction is preferred to be carried out with raw data and CNN models designed with the right parameters. The developed model is shown in Figure 3.

The proposed model starts the training with the batch normalization layer and then data enters 3 consecutive blocks. Each block consists of 3 × (convolution–batch normalization), separable convolution, batch normalization, Elu activation function, average pooling and dropout layers, respectively. After these three blocks, the data enters the flatten layer and dense layer and then is ready for classification.

Since we will start training with raw data in the model we have developed, the data first enters the batch normalization layer to be normalized. Normalized data enters the convolutional layer to extract temporal, spatial or spatiotemporal features according to the input shape structure. Here, the kernel size for 2D and 3D input shapes is [30 × 1] and the number of filters is 64. For the batch normalization layers, the momentum is 0.1 and the epsilon value is 1 × 10^−5^. After each triple convolution–batch normalization sequence, the data enters the separable convolution layer. In this layer, kernel operations are performed by factorizing the convolutional kernel into two smaller kernels and then the data is normalized. For the separable convolutional layer, the kernel size is [15 × 1] and the number of filters is 64. The Elu activation function is then applied.

The Elu function produces more accurate results by converging cost to zero faster. Its expression is as follows:

(4) $$R\left(z\right)=\left\{\begin{array}{cc} z, & z>0\\ \alpha \cdot \left(e^{z}-1\right), & z\leq 0 \end{array}\right.$$

where *α* is a constant between 0 and 1 and defined by the user.

The data enters the average pooling layer to reduce the number of model parameters and perform faster operations by acquiring new features. In this layer, the data is resized by reducing it by ½. Afterwards, 70% of the data is randomly separated with the dropout layer, preventing the overfitting tendency of the model. The data coming out of the dropout layer enter the second block to pass through the same sequence.

In this block, the kernel size for the convolutional layers is [15 × 1] and the number of filters is 32. For the separable convolutional layer, the kernel size is [7 × 1] and the number of filters is 32. Batch normalization, activation function, average pooling and dropout parameters are the same as the first block. In the third block, the kernel size for the convolutional layers is [15 × 1] and the number of filters is 16. For the separable convolutional layer, the kernel size is [7 × 1] and the number of filters is 16. Batch normalization, activation function, average pooling and dropout parameters are the same as other blocks. After the data is processed in three blocks, it enters the flatten layer and the dense layer, and then the SoftMax activation function is applied.

## 3. Experimental Results

Python programming language, Tensorflow and Keras libraries were used to design the training and classification processes covering the datasets, input shapes and CNN models defined in the previous sections. GeForce RTX 2080 Super GPU card, 32 GB ram and 12 core 3.80 GHz processor were used as hardware. During the training of the datasets, only the training sets were used, and the test sets were not included in the training in any way. Training sets are divided into two parts, 80% training and 20% validation. Determining the validation set allows us to investigate the tendency of the model to be overfitting or underfitting during training. The early stopping method was used to prevent overfitting. In this method, the validation loss value is chosen as a variable and the loss value is recorded after each epoch. If the validation loss value does not decrease during the following 20 epochs, the training session is finished and the model with the best value is taken. With this model, the performance of the model was measured with a test set that had never been seen before. Accuracy values were measured for each subject according to each input shape, and their average accuracy values were calculated according to the input shapes.

In this section, training and classification of BCI-IV-2A and BCI-IV-2B datasets was performed by using eight different input shapes and the two CNN models we designed. Training and validation accuracy graphs, training and loss values graphs and epoch time values were obtained; after training, accuracy values for each subject, their average values and confusion matrices were obtained and statistical values of models were given.

### 3.1. Training and Validation Graphs

**Figure 4:**
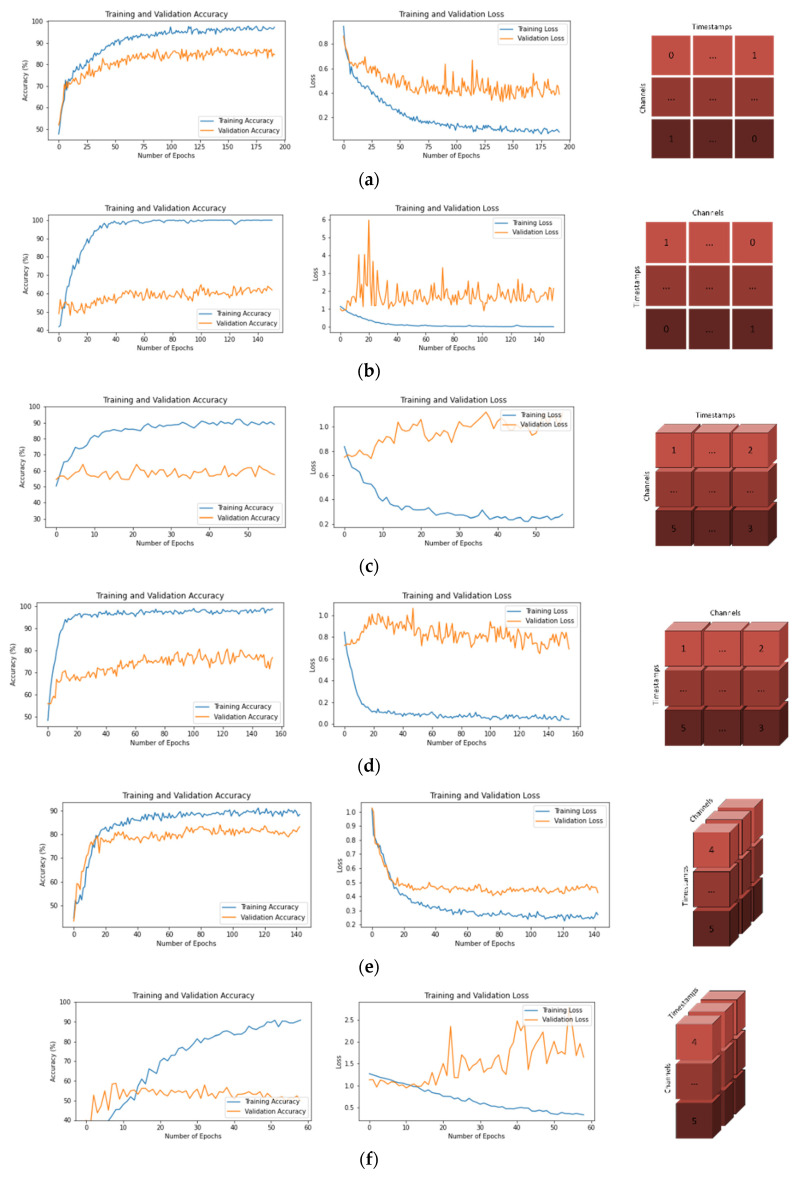

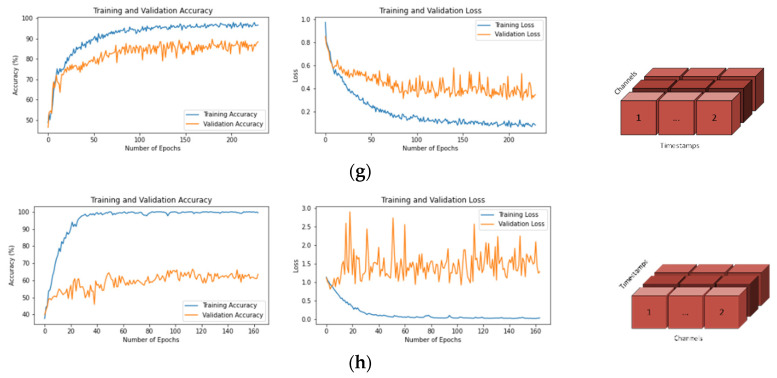
Training—Validation Accuracies and Losses for BCI-IV-2A Dataset and Input Shapes. (**a**) T × C, (**b**) C × T, (**c**) T × C × 1, (**d**) C × T × 1, (**e**) 1 × T × C, (**f**) 1 × C × T, (**g**) T × 1 × C, (**h**) C × 1 × T.

**Figure 5:**
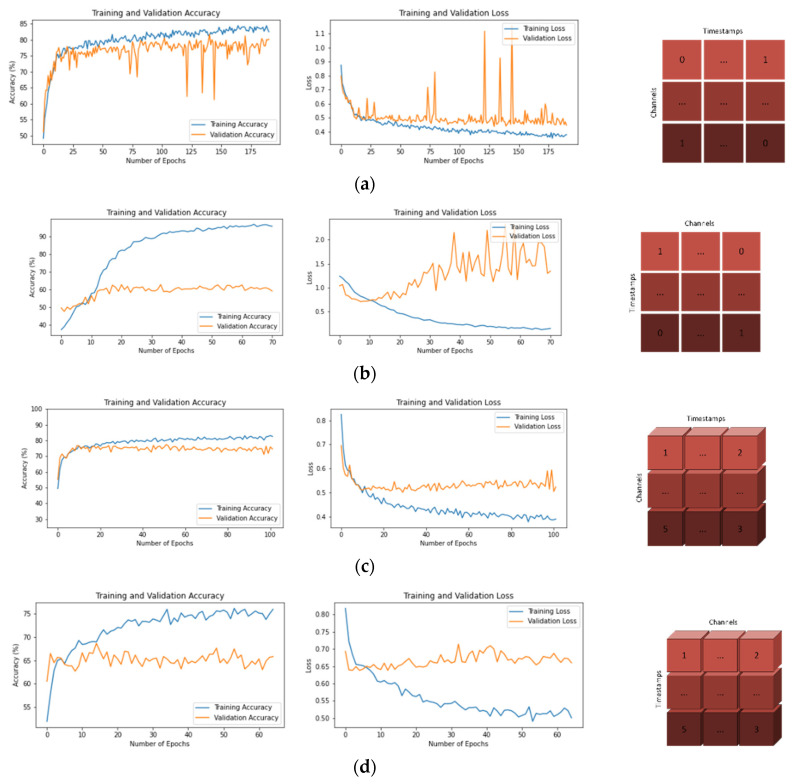

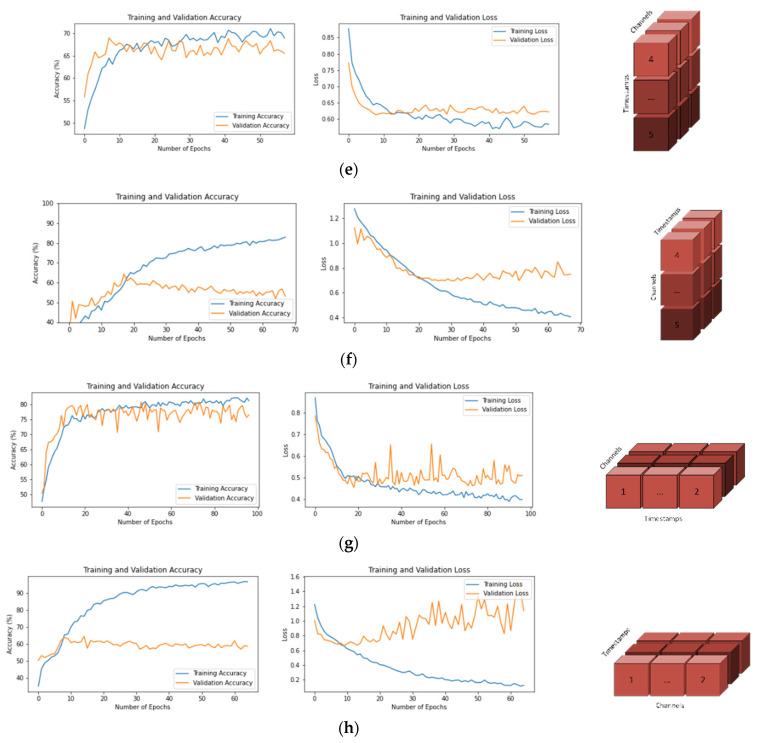
Training—Validation Accuracies and Losses for BCI-IV-2B Dataset and Input Shapes. (**a**) T × C, (**b**) C × T, (**c**) T × C × 1, (**d**) C × T × 1, (**e**) 1 × T × C, (**f**) 1 × C × T, (**g**) T × 1 × C, (**h**) C × 1 × T.

While both datasets were trained with the developed CNN models and eight different input shapes, training–validation accuracy graphs and training–validation loss graphs were obtained in order to better observe and analyze the training process. These graphs are shown in Figure 4 for the BCI-IV-2A dataset and in Figure 5 for the BCI-IV-2B dataset.

### 3.2. Accuracy Values

In order to measure the effect of the designed input shape structures on the success of the classifier, the subject-based and average accuracy values of each input shape were calculated. While calculating these values, we used the test set, which is a separate group in the dataset that had not been used in the training phase or seen by the model before. The obtained accuracy values are given in Table 1 for the BCI-IV-2A dataset and in Table 2 for the BCI-IV-2B dataset.

### 3.3. Epoch Times

As a result of the training for each input shape, the training epoch times were measured for the datasets used. Epoch times are seconds measured for a single epoch. The measured epoch times for the BCI-IV-2A and BCI-IV-2B datasets are given in Table 3.

### 3.4. Confusion Matrices

Confusion matrix graphs were created to measure the ability of the trained models to distinguish the classes in the dataset and to determine which classes can be better distinguished with which input shape. With these graphs, true positive, true negative, false positive and false negative numbers and ratios of the models were obtained. These obtained values are given in Table 4 for the BCI-IV-2A and BCI-IV-2B datasets.

### 3.5. Model Statistics

The statistical values of the models were calculated according to the input shape structures for the datasets. The F1 score, which is the harmonic mean of the precision and recall values and the success of balancing the precision and recall values of the model, the cappa coefficient value and the standard deviation values for each input shape were obtained. These obtained values are given in Table 5 for both datasets.

## 4. Discussion

In this study, the datasets used were converted into 2D and 3D matrix sizes, and these conversion processes provided a total of eight different input shapes to be obtained; two different combinations in 2D structure and six different combinations in 3D structure. Subsequently, 2D and 3D CNN models were created to train 2D and 3D input shape structures. These models have the same type of layers, same number of layers and the same parameters. The raw data prepared in the input shape structures described in the previous sections were given as direct input to the designed CNN models without any signal preprocessing.

In this section, the results obtained during and after training the datasets with eight different input shape structures were compared with each other, the pros and cons of the models were presented, and the results obtained were evaluated.

**Figure 6:**
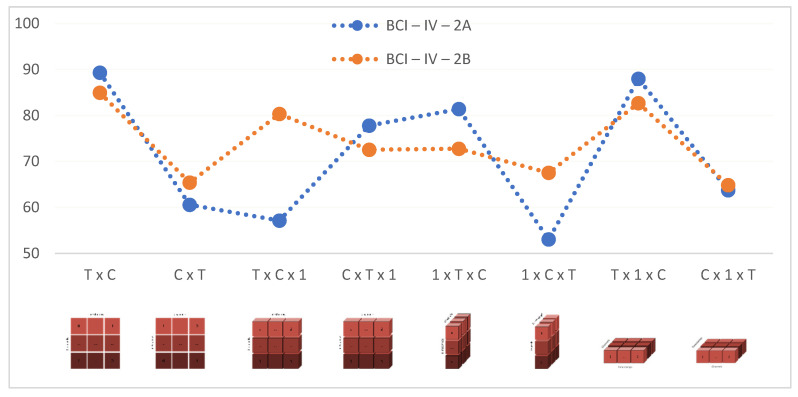
Average Accuracy Values for each Input Shape.

The models trained with raw data, without applying any preprocessing methods, showed a classification success between 53.03% and 89.29% for the BCI-IV-2A dataset and between 65.38% and 84.94% for the BCI-IV-2B dataset. Classification accuracies according to the input shape change are shown in Figure 6.

**Figure 7:**
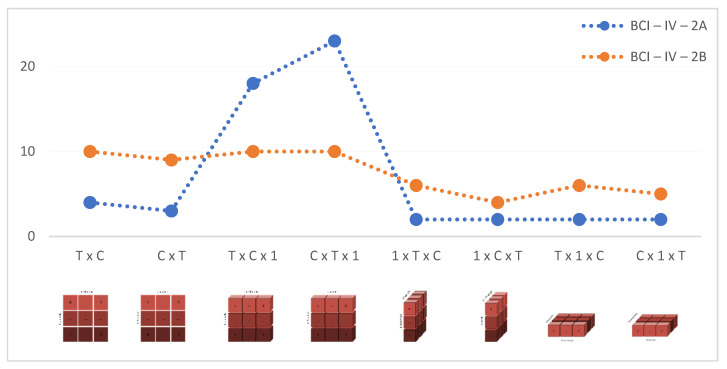
Epoch Times for each Input Shape.

In addition to these, it has been observed that the different input shapes and the number of channels in the dataset are also effective on the epoch times. Epoch times were measured in the range of 2–23 s for BCI-IV-2A and 4–10 s for BCI-IV-2B. The epoch times according to the input shape change are shown in Figure 7.

With the input shape in the T × C structure, which achieved the best accuracy for the BCI-IV-2A dataset, an average accuracy rate of 89.293% and a standard deviation of 5.471 were obtained. With the input shape in the T × C structure, which achieved the best accuracy for the BCI-IV-2B dataset, an average accuracy rate of 84.943% and a standard deviation of 8.607 were obtained. These values obtained with the developed models reveal the high classification success and robustness of the models.

In both datasets, the average accuracy values span a wide range (range value of 36.26 for 2A dataset and 20.10 for 2B dataset). It has been observed that some input shapes cause high levels of overfitting during training (Figure 4b,f,h, and Figure 5)b,f,h. Although these input shapes have very high training accuracy values, their validation values are quite low. Input shapes have been observed to have a significant effect on epoch times as well (Table 3). In both datasets, the epoch times are spread over a wide range (range value of 21 for dataset 2A and range value of 6 for dataset 2B). As a result, it has been determined that input shape structures are a very important parameter in the performance of CNN models (Table 1 and Table 2).

In both datasets, the highest accuracy values were achieved with T × C and T × 1 × C input shapes respectively (Figure 6). Researchers can train their models by selecting appropriate input shapes for 2D and 3D CNN architectures if needed. In both datasets, it has been observed that parallel changes in the accuracy and epoch time values are obtained as the input shape changes (Figure 6 and Figure 7). However, it cannot be concluded that a given input shape will achieve the same success in every CNN model and every dataset to be used (Figure 6). Therefore, it was concluded that the researchers should try different input shape structures for the CNN models they set up.

In the BCI-IV-2A dataset, both higher average accuracy values and lower standard deviations were obtained compared to the 2D dataset (Table 5). When the training and validation graphs were examined, it was observed that the models trained with the 2A dataset learned faster and reached lower loss values than the models trained with the 2B dataset (Figure 4 and Figure 5). The decisive reason for this is that the 2A dataset has 22 EEG channels, while the 2B dataset has 3 channels.

To deal with the possible covariate shift, we used batch normalization layers along the path of the data. Instead of normalizing all the data outside the model and giving it as input to the model, we started the model with batch normalization. In this way, each batch entering the model was normalized only within itself, not all the data together. The data was then propagated through layers. When the data enters a new layer, it is processed here and the 0 mean and unit variance distributions change. In order to avoid covariate shifts that may arise as a result of these changes, we used a batch normalization layer after each convolutional layer and ensured that the data were normalized throughout the training in the model. Since each batch entering the model may show a very different distribution from the previous or next batches, we kept the feature values of the batches on the same scale in order to achieve high classification success with a more robust and stable model against possible covariate shifts.

In models with high classification accuracy, we predict that the model establishes a relationship between different channels, since the EEG channels are scanned one by one depending on time during the training of the input shapes. In this way, we predict that higher classification accuracies will be achieved by scanning the relationships between the EEG channels of the test data entering the models, and that the models will be more stable and invariant against time-dependent feature changes. In models with low classification accuracy, we predict that the models scan channels together in a single time period during training and establishing relationships between time periods rather than between channels. In this way, we predict that models looking for relationships between certain time periods will give low test accuracy values against nonstationary EEG signals whose time-dependent properties are highly variable.

Large amounts of training data are needed to achieve high classification success using deep neural networks. However, collecting large amounts of motor imagery signals is quite challenging. Pre-training and experience are needed to collect these signals. In addition, the subjects’ inability to keep their attention at the same level during the signal acquisition session, fatigue and the presence of environmental disrupting factors make this process even more difficult.

In order to overcome this problem, researchers have used data augmentation methods that create new data and increase existing dataset systematically. Data augmentation can reduce the tendency of overfitting and increase the classification success and stability of the model. It can also enable models trained with nonstationary EEG data to make fewer mistakes, be invariant and increase robustness when encountering new test data [49,50,51]. Signal collection processes can take days, weeks and even months, and data augmentation applications have shown very promising results compared to the results obtained as a result of these signal collection processes [52,53] These gains may also reduce the time and funds that researchers allocate to collect signals [30].

Our CNN model, which was trained with raw data without signal preprocessing methods, achieved accuracy values close to or better than many state-of-the-art models with the correct input shape and correct CNN architecture [20,26,42,54,55,56,57,58,59,60]. Accordingly, it has been shown that by establishing the right CNN architecture and choosing the right input shape structure, feature extraction of EEG motor imagery signals can be done successfully with raw data. Thus, the loss of possible distinguishing features in sensitive EEG signals is prevented and much faster and more successful results can be obtained by saving time and effort.

## 5. Conclusions

In this study, the effect of input shape structures on the performance of CNN models used in the classification of EEG motor imagery signals is shown with quantitative data. The obtained results showed that even if researchers develop correct CNN models during the classification of EEG signals, if they choose the inaccurate input shape, they can achieve poor classification success. This may cause researchers to disrupt the correct models they have established, which may increase the time and energy they will spend to build a model. In order to prevent this situation, researchers can increase the classifier performance by trying different input shape structures and minimize the losses they may experience due to this parameter.

Training and classification with raw signals can let researchers develop real-time, fast, reliable and robust models with high accuracy values. Since signal preprocessing methods are not used, possible data loss that may occur in these processes can be prevented. In addition, pre-training processes can be shortened by automating feature extraction processes. Along with these advantages, there are also some disadvantages. Deep learning models need a large amount of data, and the difficulty of collecting EEG signals may lead to the need to develop data augmentation methods. In addition, hyperparameter optimization may be required for newly designed neural network models to achieve high classification success, which may cause time and computational costs.

These advantages and disadvantages create some future research opportunities. Increases in classification performance can be achieved quickly by applying fine-tuning and hyperparameter optimization to deep learning models, which are previously designed and used by researchers. In addition, new neural network layers and architectures can be designed to detect EEG features accurately and quickly, and the right parameters for models can be searched. By developing data augmentation methods, it is possible to obtain the required large amount of data. Furthermore, previously or newly developed robust and fast models can be used and tested in real-time systems.

In addition to all these, with a correctly constructed CNN model, high classification success can be achieved without the need for labor-intensive and time-consuming signal preprocessing to extract distinctive features from EEG signals. With the CNN model, which was developed in this study and uses raw data, close or better results were obtained than the state-of-the-art models that achieved high classification success using signal preprocessing.

It is predicted that this developed methodology will be useful in the development and use of real-time brain–computer interfaces as it does not need signal preprocessing, can train the system quickly with new data, and is robust with low standard deviation values.

## Figures and Tables

**Table 1:** Subject-Based and Average Accuracy Values of BCI-IV-2A.

	T × C	C × T	T × C × 1	C × T × 1	1 × T × C	1 × C × T	T × 1 × C	C × 1 × T
S1	84.40	60.28	54.61	63.12	68.09	47.52	81.56	60.28
S2	78.87	54.23	51.41	72.54	80.28	51.41	81.69	58.45
S3	96.35	54.74	48.18	71.53	75.18	51.82	88.32	59.12
S4	88.79	61.21	60.34	79.31	81.90	51.72	84.48	64.66
S5	92.59	72.59	68.15	87.41	94.07	57.04	96.30	74.81
S6	84.26	59.26	56.48	74.07	80.56	50.00	83.33	62.04
S7	92.14	72.14	65.00	91.43	85.71	56.43	93.57	74.29
S8	97.76	55.97	55.97	79.10	84.33	55.22	94.03	59.70
S9	88.46	54.62	53.85	81.54	82.31	56.15	88.46	60.00
Average	89.29	60.56	57.11	77.78	81.38	53.03	87.97	63.71
STD	5.47	6.41	5.70	7.70	6.38	2.96	5.02	5.74

**Table 2:** Subject-Based and Average Accuracy Values of BCI-IV-2B.

	T × C	C × T	T × C × 1	C × T × 1	1 × T × C	1 × C × T	T × 1 × C	C × 1 × T
S1	78.95	62.72	70.61	64.91	63.16	59.65	78.95	61.40
S2	70.20	56.73	63.67	71.43	64.49	66.53	64.49	61.63
S3	73.48	60.87	67.39	68.70	76.09	67.83	66.96	58.70
S4	96.74	57.33	94.14	64.17	61.56	58.96	94.79	58.31
S5	96.70	93.04	92.31	95.24	97.07	93.77	98.53	89.01
S6	83.67	60.96	76.10	69.32	68.53	64.14	78.09	61.35
S7	90.09	80.60	87.93	81.47	89.66	82.76	93.10	76.72
S8	91.74	64.35	88.26	81.30	78.26	62.61	89.57	62.17
S9	82.86	51.84	82.45	56.33	55.92	51.43	79.59	54.29
Average	84.94	65.38	80.32	72.54	72.75	67.52	82.67	64.84
STD	8.61	11.70	10.08	10.44	12.27	11.63	10.85	9.79

**Table 3:** Epoch times for each input shape for BCI-IV-2A and BCI-IV-2B.

Input Shape	Epoch Time (Second/Epoch)	
	BCI-IV-2A	BCI-IV-2B
T × C	4	10
C × T	3	9
T × C × 1	18	10
C × T × 1	23	10
1 × T × C	2	6
1 × C × T	2	4
T × 1 × C	2	6
C × 1 × T	2	5

**Table 4:** Confusion Matrix Values for BCI-IV-2A and BCI-IV-2B.

Input Shape	BCI-IV-2A				BCI-IV-2B			
	TL	FL	TR	FR	TL	FL	TR	FR
T × C	536/593 (90.4%)	57	521/590	69	936/1118 (83.7%)	182	979/1123	144
		(9.6%)	(88.3%)	(11.7%)		(16.3%)	(87.2%)	(12.8%)
C × T	384/593 (64.8%)	209	333/590	257	665/1118 (59.5%)	453	802/1123	321
		(35.2%)	(56.4%)	(43.6%)		(40.5%)	(71.4%)	(28.6%)
T × C × 1	314/593 (53.0%)	279	361/590	229	931/1118 (83.3%)	187	882/1123	241
		(47.0%)	(61.2%)	(38.8%)		(16.7%)	(78.5%)	(21.5%)
C × T × 1	440/593 (74.2%)	153	480/590	110	749/1118 (67.0%)	369	877/1123	246
		(25.8%)	(81.4%)	(18.6%)		(33.0%)	(78.1%)	(21.9%)
1 × T × C	481/593 (81.1%)	112	481/590	109	805/1118 (72.0%)	313	823/1123	300
		(18.9%)	(81.5%)	(18.5%)		(28.0%)	(73.3%)	(26.7%)
1 × C × T	329/593 (55.5%)	264	299/590	291	675/1118 (60.4%)	443	840/1123	283
		(44.5%)	(50.7%)	(49.3%)		(39.6%)	(74.8%)	(25.2%)
T × 1 × C	514/593 (86.7%)	79	528/590	62	876/1118 (78.4%)	242	989/1123	134
		(13.3%)	(89.5%)	(10.5%)		(21.6%)	(88.1%)	(11.9%)
C × 1 × T	378/593 (63.7%)	215	376/590	214	610/1118 (54.6%)	508	846/1123	277
		(36.3%)	(63.7%)	(36.3%)		(45.4%)	(75.3%)	(24.7%)

TL: True Left; FL: False Left; TR: True Right; FR: False Right.

**Table 5:** Statistical Values of BCI-IV-2A and BCI-IV-2B Models.

Input Shape	BCI-IV-2A				BCI-IV-2B			
	Accuracy	F1 Score	Kappa	STD	Accuracy	F1 Score	Kappa	STD
T × C	89.293	0.893	0.787	5.471	84.943	0.854	0.709	8.607
C × T	60.561	0.605	0.212	6.413	65.383	0.653	0.309	11.695
T × C × 1	57.114	0.570	0.141	5.704	80.318	0.809	0.618	10.083
C × T × 1	77.782	0.777	0.555	7.697	72.538	0.725	0.451	10.436
1 × T × C	81.376	0.813	0.626	6.376	72.747	0.726	0.453	12.269
1 × C × T	53.033	0.531	0.062	2.958	67.518	0.674	0.352	11.628
T × 1 × C	87.969	0.881	0.762	5.021	82.665	0.830	0.664	10.846
C × 1 × T	63.713	0.637	0.275	5.737	64.835	0.646	0.299	9.790

## Data Availability

Publicly available BCI Competition IV 2A and BCI Competition IV 2B datasets were used in this research and these can be accessed via https://www.bbci.de/competition/iv/.
